# Supplementary figures and images for: Allosteric modulation of proton binding confers Cl- activation and glutamate selectivity to vesicular glutamate transporters
Source: PLoS Comput Biol. 2025 Jun 26;21(6):e1013214. doi: 10.1371/journal.pcbi.1013214 (PMC12240346; doi:10.1371/journal.pcbi.1013214)

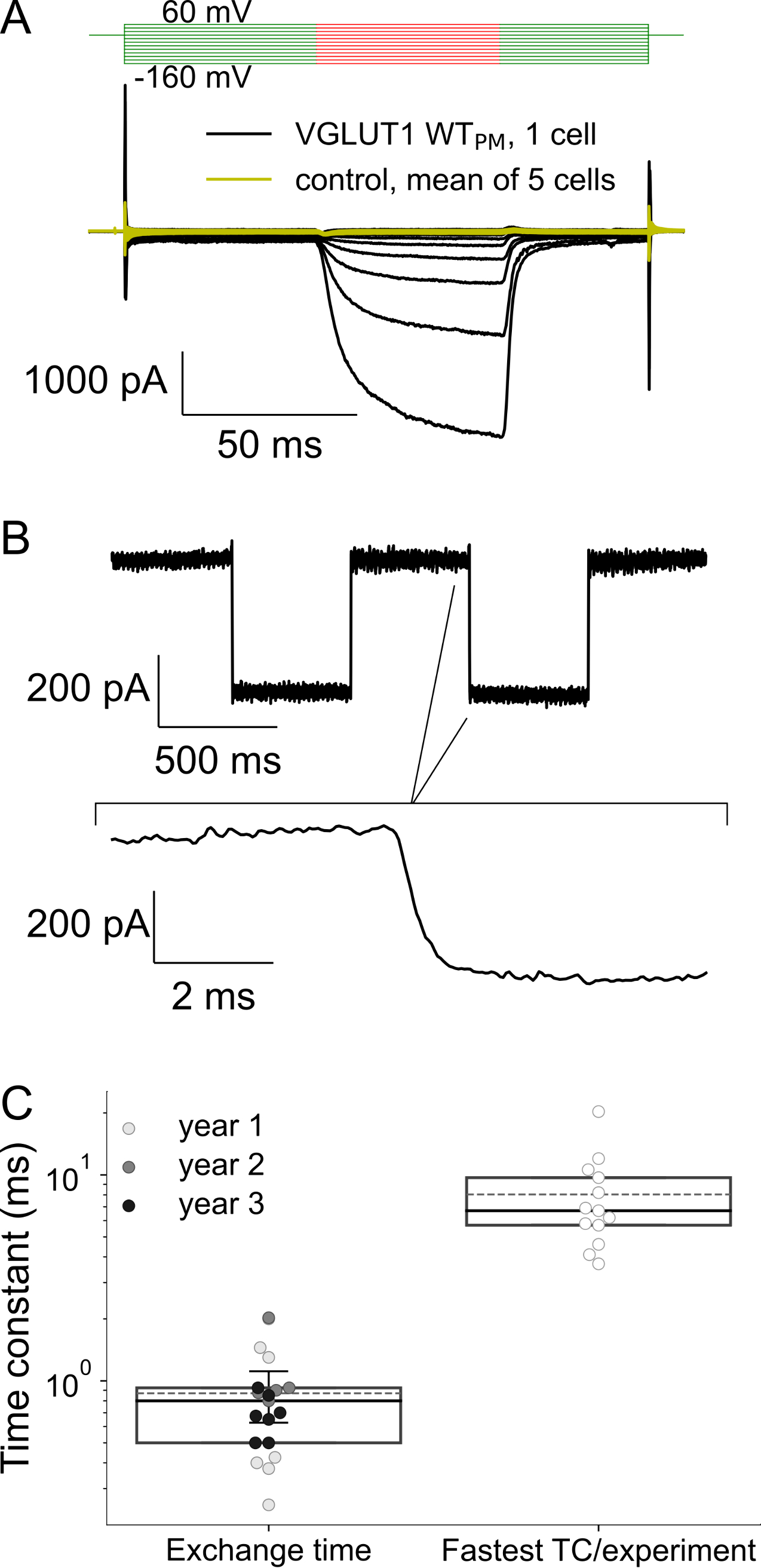

Supplement: S1 Fig — (A) Mean Cl- current responses to pH jumps for 5 untransfected HEK293T cells (yellow), in comparison to a representative HEK293T cell expressing VGLUT1PM under identical conditions (black). The pH jumps are from pH 7.4 to 5.5 and back, at -160–60 mV and with 140 mM external Cl-. (B) Representative recording of the protocol used to test open pipette solution exchange time, typically between different [Cl-]. (C) Calculated exchange times collected across several years compared to the fastest rate of all solution exchange experiments. (TIF) [file pcbi.1013214.s001.tif]

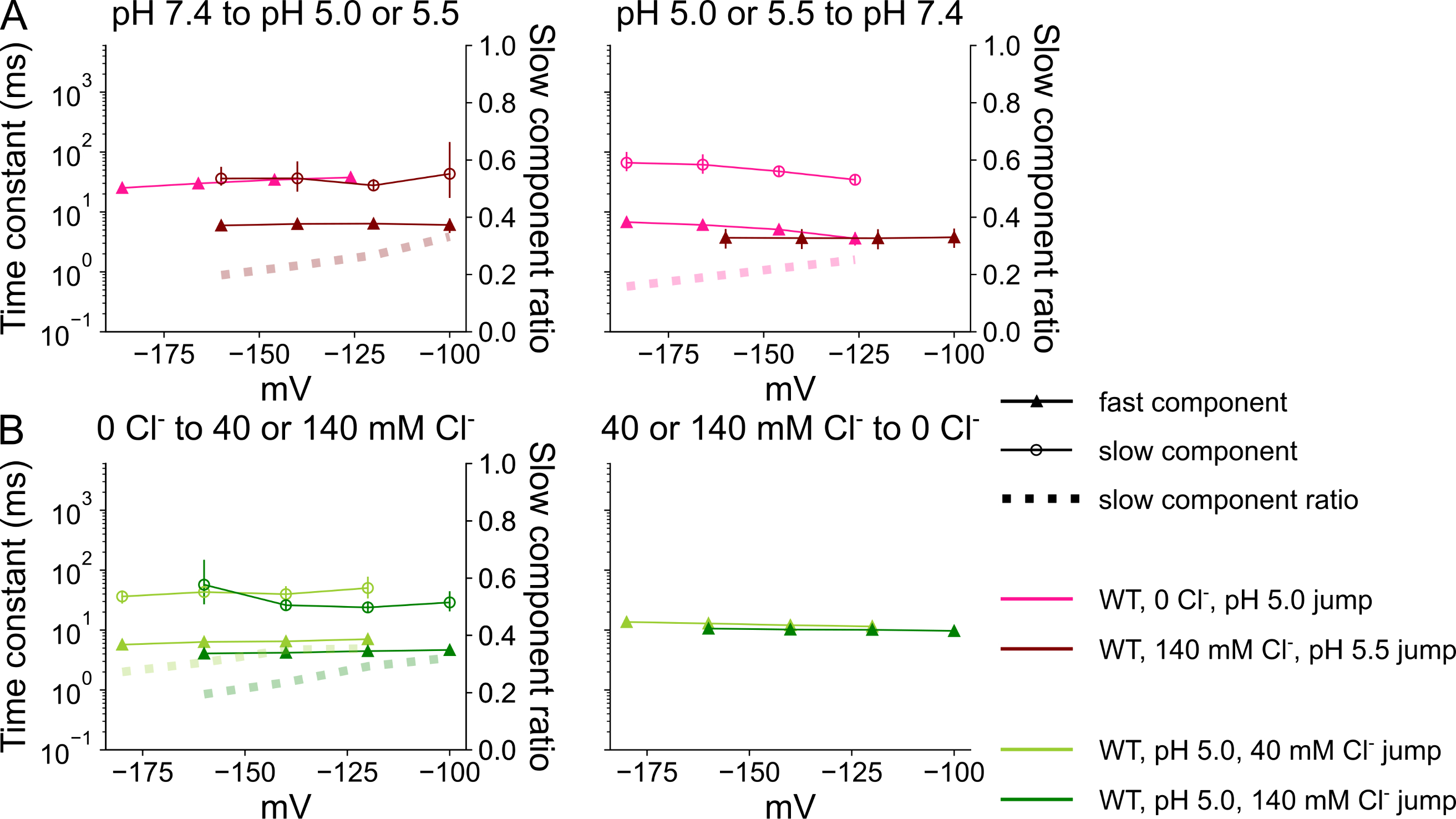

Supplement: S2 Fig — (A) activation/deactivation time constants upon pH jumps from 7.4 to 5.0 or 5.5 (left) or from 5.0 or 5.5 to 7.4 (right) at an external [Cl-] of 0 or 140 mM. (B) time constants upon [Cl-] steps from 0 to 40 mM or 0–140 mM at pH 5.5. Activation at high external Cl- or deactivation without Cl- were fitted with biexponential functions, providing two time constants and the relative amplitude (dashed lines) of the slower component. Data are shown as means obtained by bootstrapping with a global fit of experimental data with a sampling of 1000, with 95% of the sampling as error bars. Voltage differences are the result of a posteriori liquid junction potential correction. (TIF) [file pcbi.1013214.s002.tif]

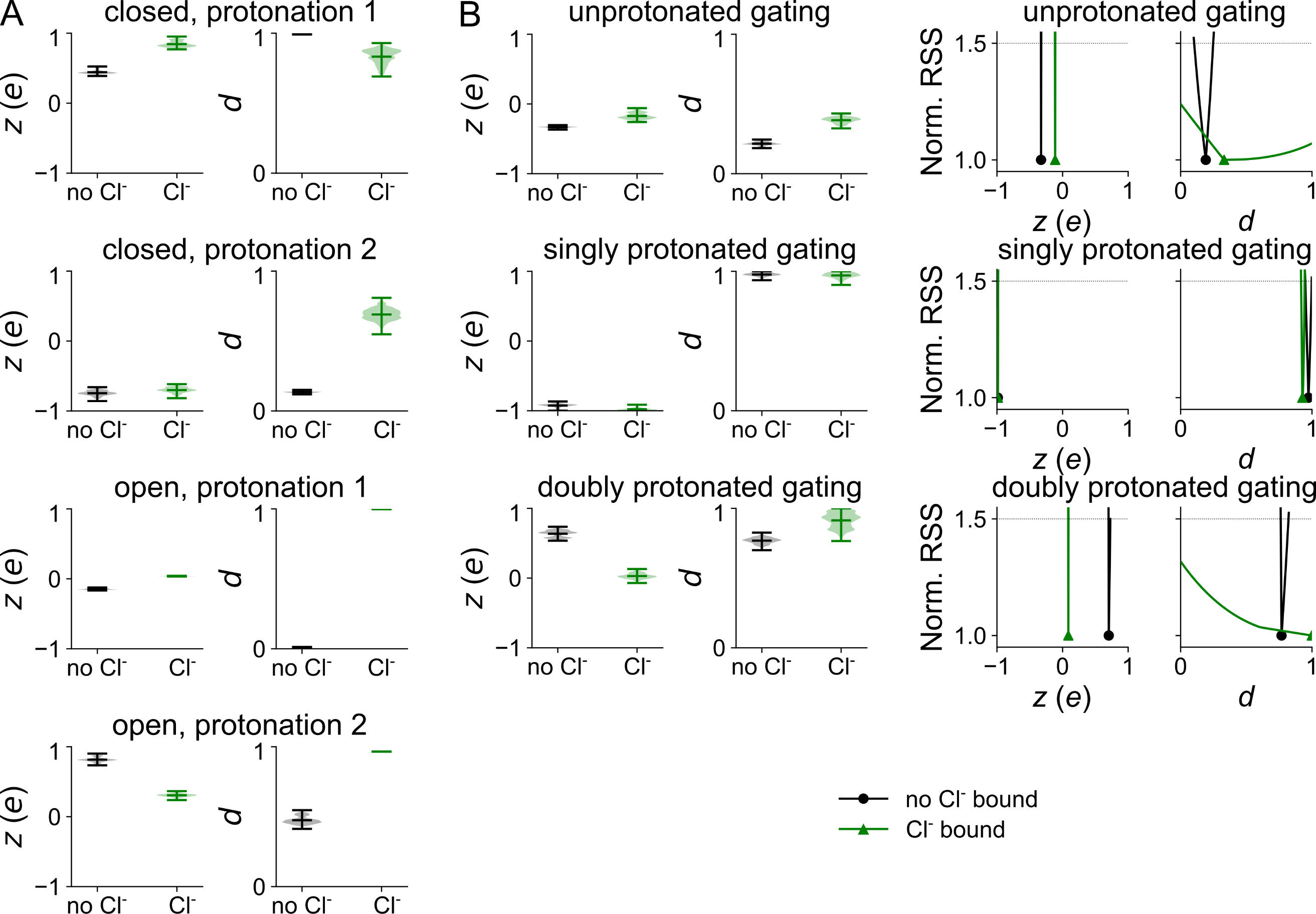

Supplement: S3 Fig — (A) distribution of z and d parameters for protonation steps with and without Cl-. (B) distribution of z and d parameters for channel opening. Protonation parameters are represented by violin plots, other simulation results are given as normalized RSS representing goodness of fit for a range of amplitudes in addition to violin plots depicting the amplitude range generated by exploratory mutation. (TIF) [file pcbi.1013214.s003.tif]

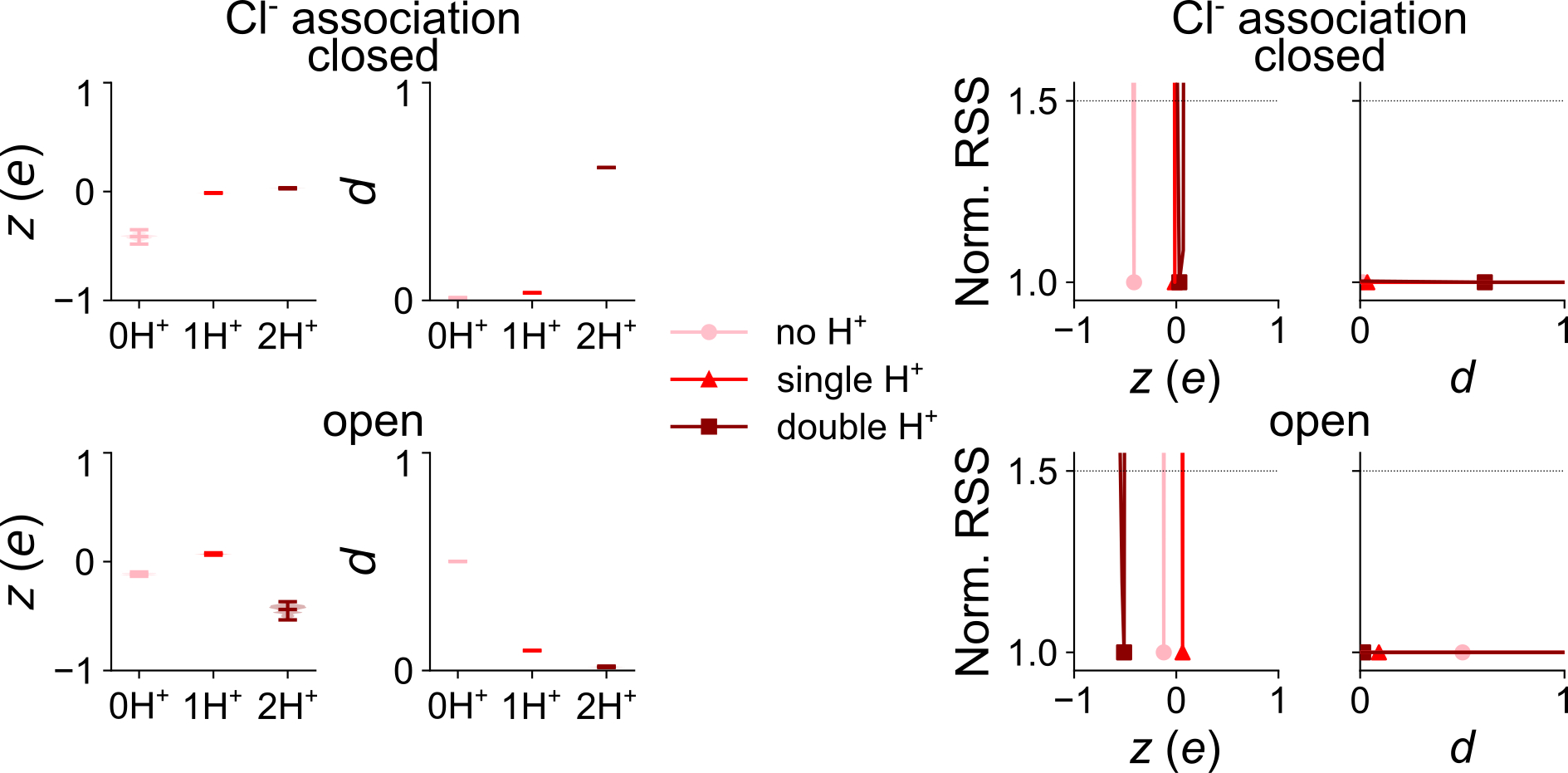

Supplement: S4 Fig — Distribution of z and d parameters for the three protonation states (light→dark red indicates increasing protonation). Simulation results are given as violin plots depicting the amplitude range generated by exploratory mutation and normalized RSS representing goodness of fit for a range of amplitudes. (TIF) [file pcbi.1013214.s004.tif]

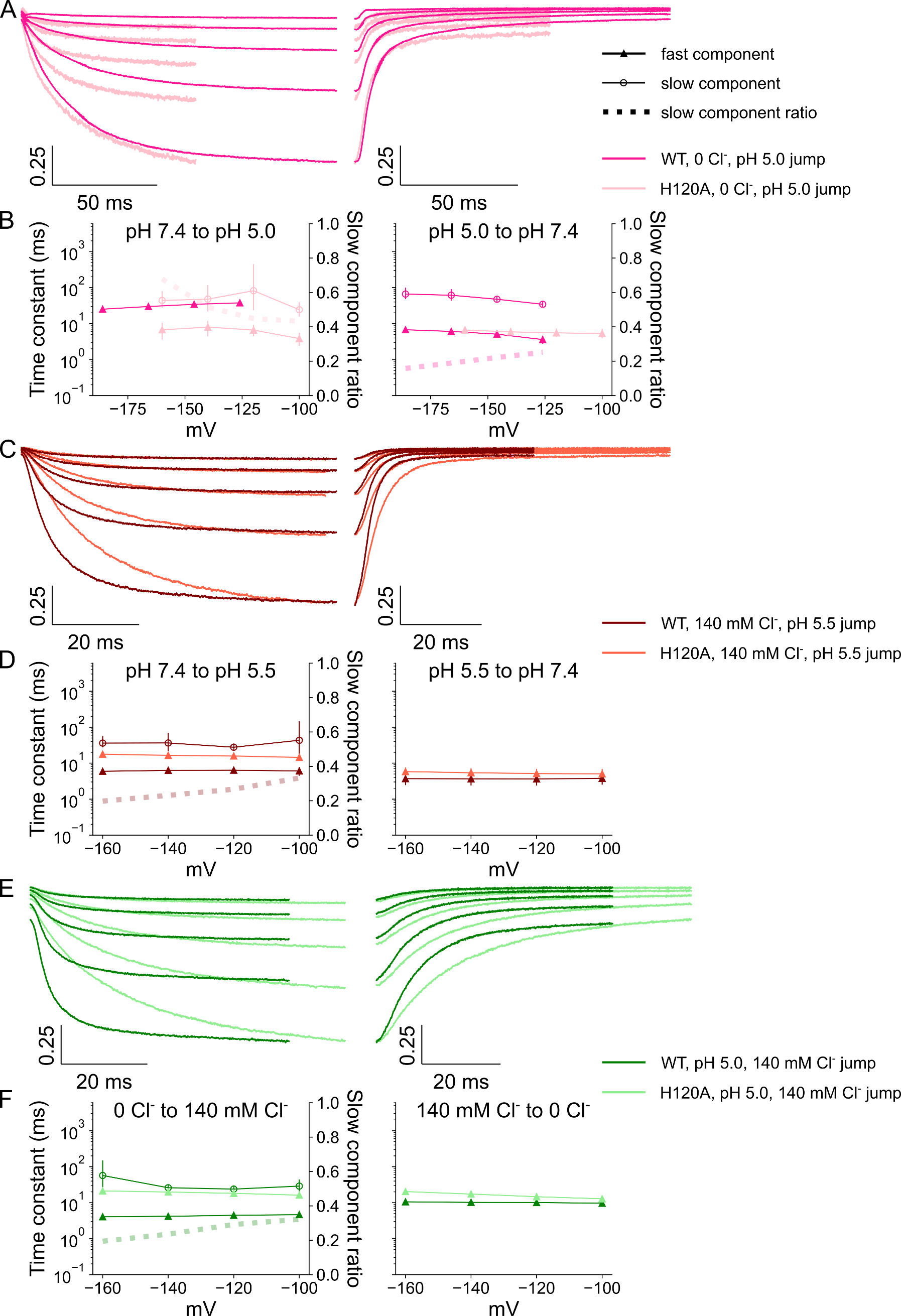

Supplement: S5 Fig — (A) representative current responses to concentration jumps from pH 7.4 to 5.0 at [Cl-] = 0 mM. (B) corresponding time constants for activation (left) and deactivation (right). (C) representative current responses to concentration jumps from pH 7.4 to 5.0 at [Cl-] = 0 mM140 mM. (D) corresponding time constants for activation (left) and deactivation (right). (E) representative current responses to concentration jumps from [Cl-] = 0–140 mM at pH 5.5. (F) corresponding time constants for activation (left) and deactivation (right) at given holding potentials. Activation at high external Cl- or deactivation without Cl- were fitted with biexponential functions, with two time constants and the relative amplitudes (dashed lines) of the slower component shown. Data are shown as means obtained by bootstrapping with a global fit of experimental data with a sampling of 1000, with 95% of the sampling as error bars. Voltage differences are the result of a posteriori liquid junction potential correction. (TIF) [file pcbi.1013214.s005.tif]

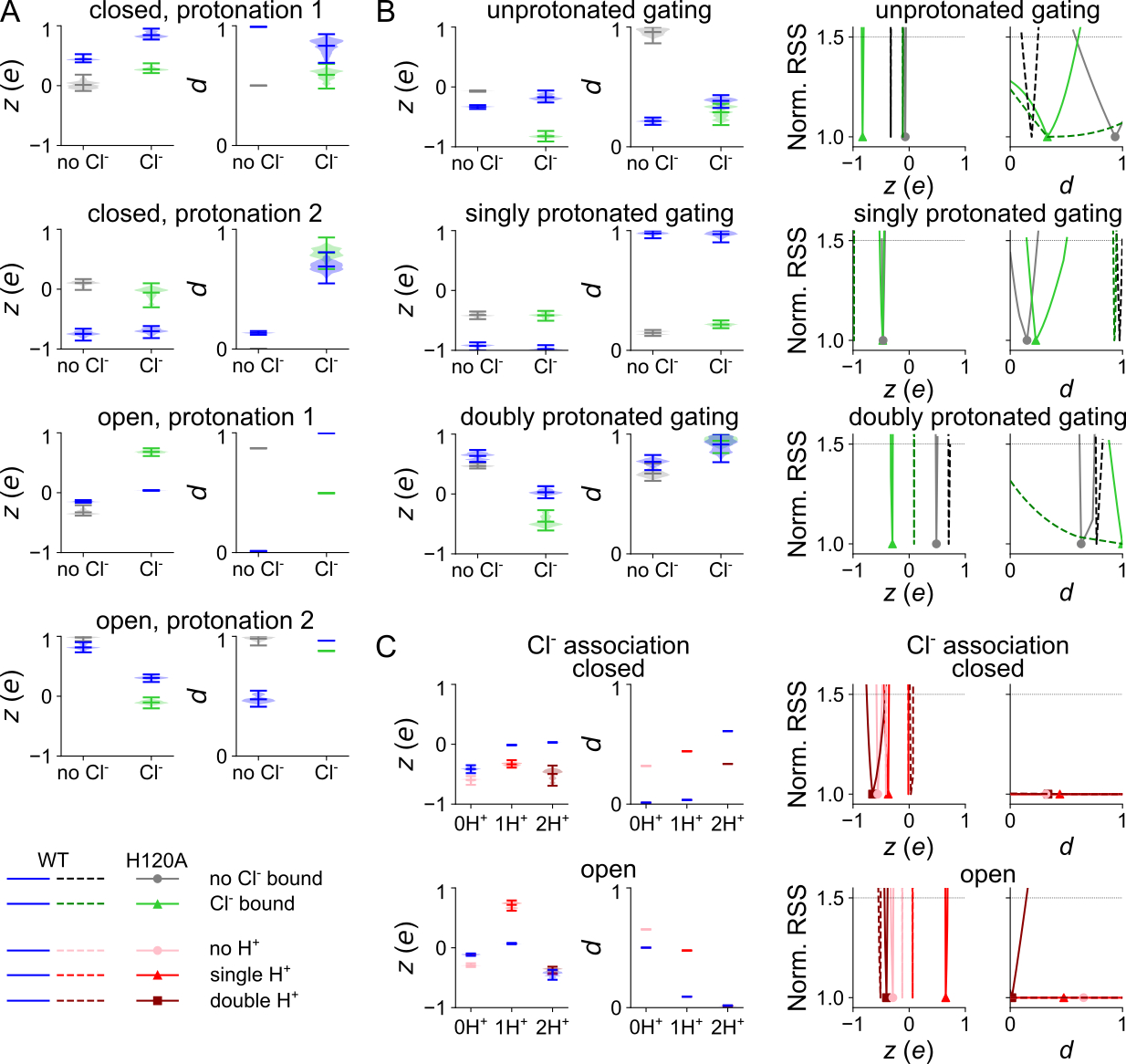

Supplement: S6 Fig — (A) distribution of z and d parameters for protonation steps with and without Cl-. (B) distribution for channel opening with and without Cl-. (C) distribution for Cl- binding by protonation state. Protonation parameters are represented by violin plots, other simulation results are given as normalized RSS representing goodness of fit for a range of amplitudes in addition to violin plots depicting the amplitude range generated by exploratory mutation. (TIF) [file pcbi.1013214.s006.tif]

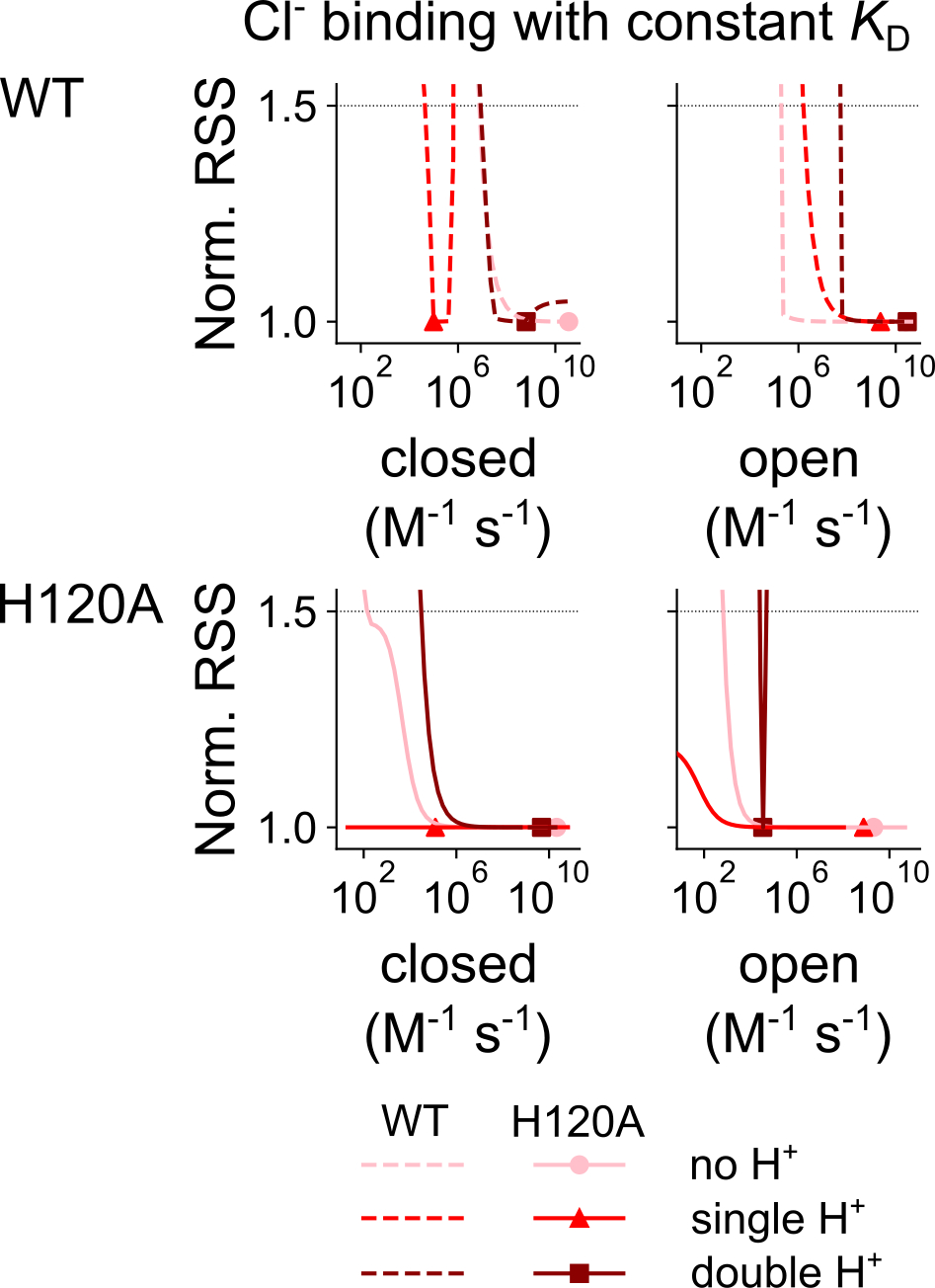

Supplement: S7 Fig — Changes in the goodness of fit upon the modification of secondary Cl--binding rate constants at -160 mV, to closed (left) or open (right) WT or H120A VGLUT1PM anion channels in the unprotonated or singly or doubly protonated state. During modification, unbinding constants were simultaneously altered to keep the Cl--binding affinity unchanged. (TIF) [file pcbi.1013214.s007.tif]

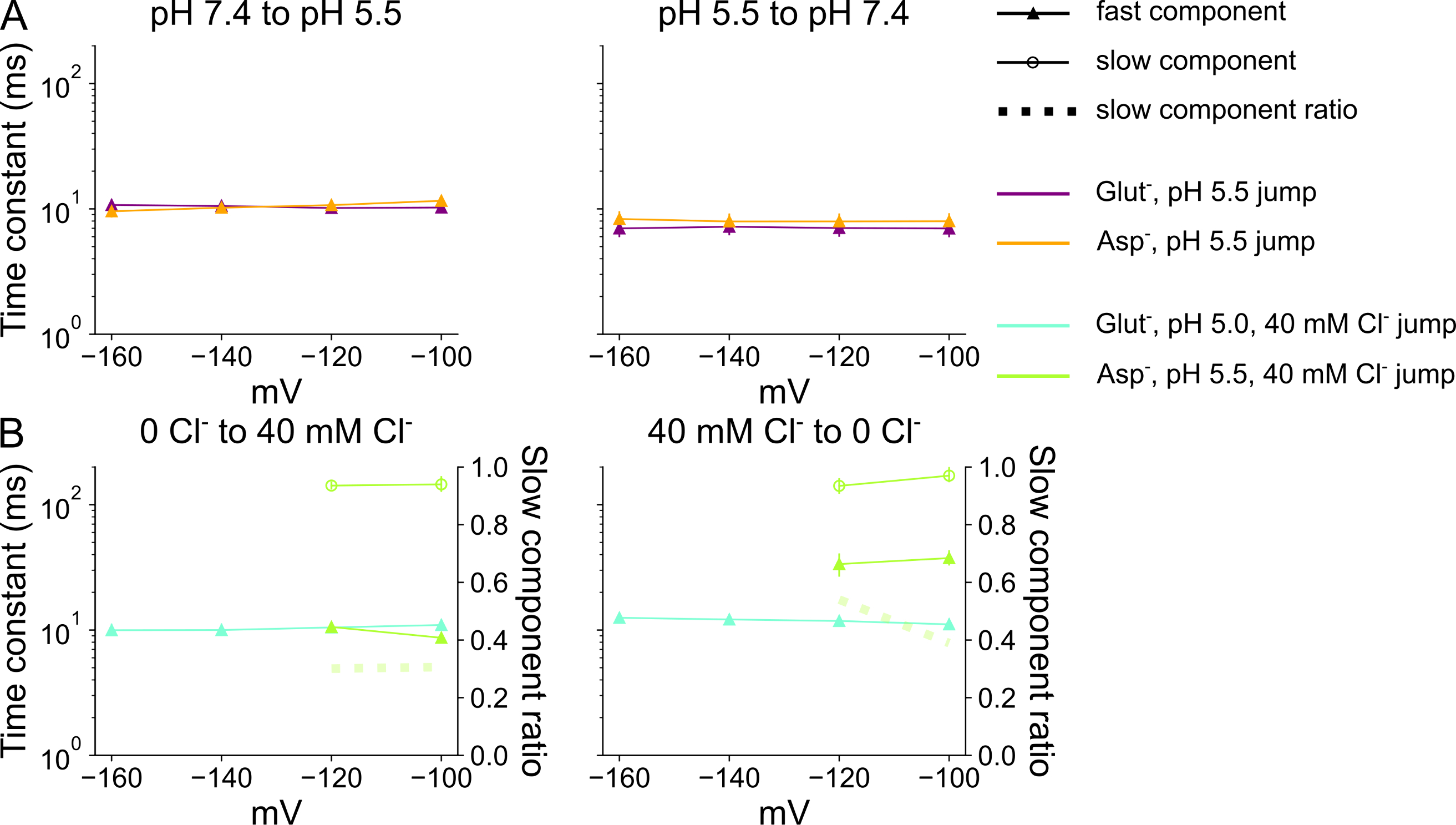

Supplement: S8 Fig — Activation/deactivation time constants upon pH jumps from 7.4 to 5.0 (left) or deactivation time constants upon pH jumps from 5.0 to 7.4 (right) at an external [Cl-] of 40 mM (A) or upon [Cl-] jumps from 0 to 40 mM (left) or deactivation time constants upon [Cl-] jumps from 40 to 0 mM (right) at an external pH of 5.0 for glutamate or 5.5 for aspartate (B). Activation and deactivation of aspartate currents by Cl- were fitted with biexponential functions, providing two time constants and relative amplitudes (dashed lines) for the slower component. Data are shown as means obtained by bootstrapping with a global fit of experimental data with a sampling of 1000, with 95% of the sampling as error bars. (TIF) [file pcbi.1013214.s008.tif]

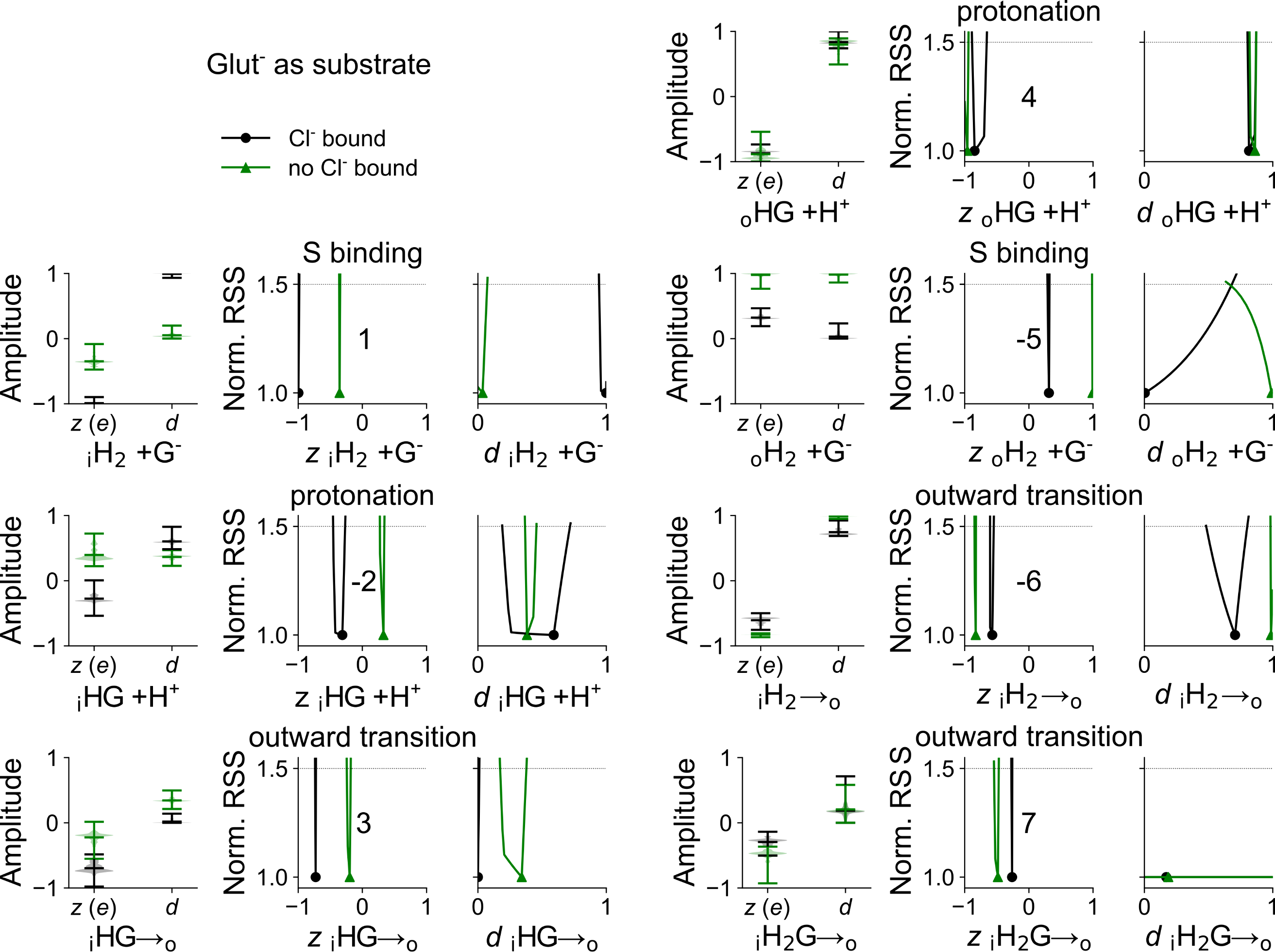

Supplement: S9 Fig — Distribution of z and d parameters for steps describing substrate binding ([1] and [5]), protonation ([2] and [4]), and transition between inward- and outward-facing conformations [3,6,7]. Simulation results are given as violin plots depicting the amplitude range generated by exploratory mutation and normalized RSS representing goodness of fit for a range of amplitudes. (TIF) [file pcbi.1013214.s009.tif]

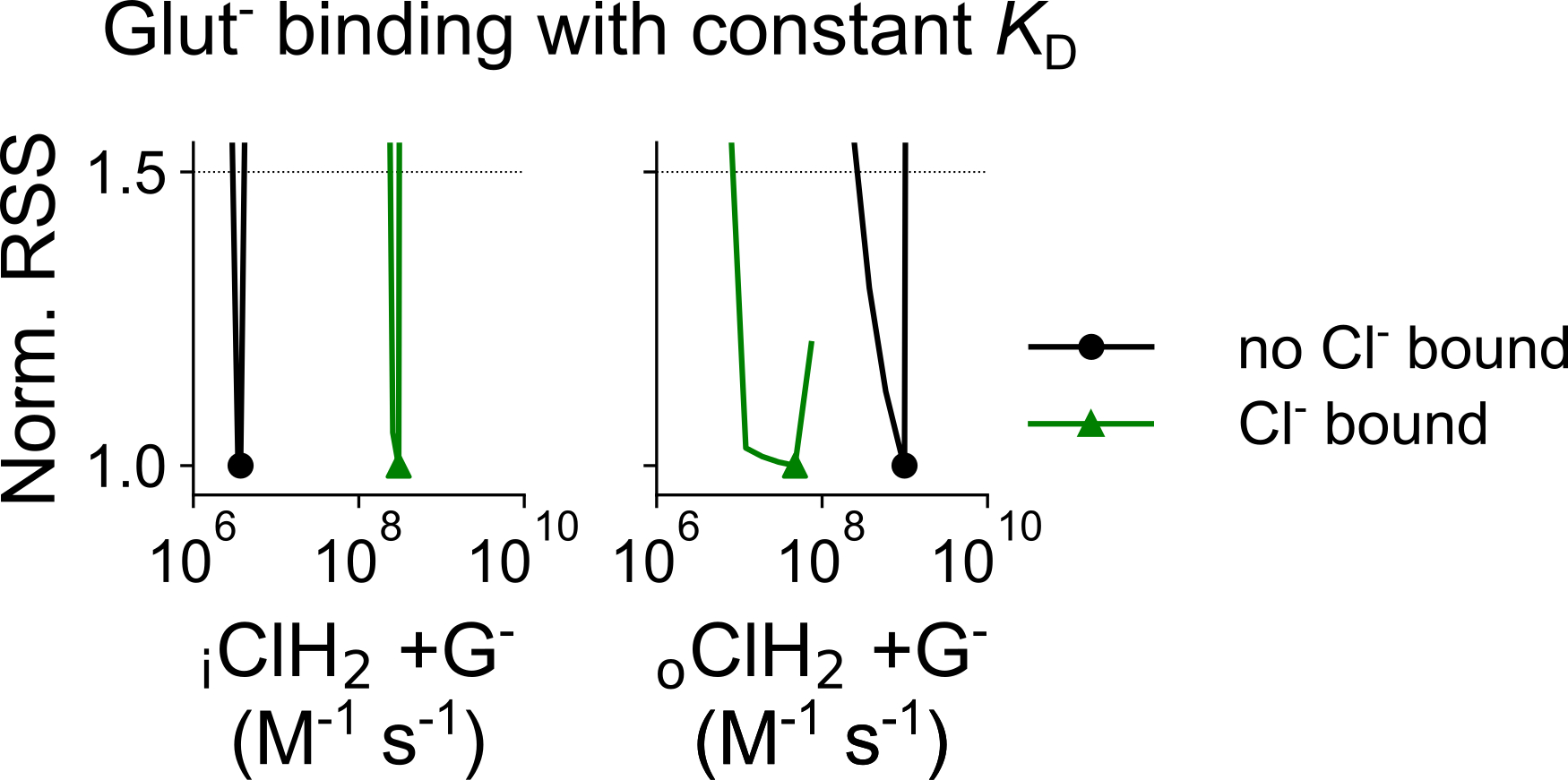

Supplement: S10 Fig — A, Changes in the goodness of fit upon modification of the binding rate for glutamate with and without Cl-, in the inward- and outward-facing conformation. During modification, the unbinding constants were simultaneously altered to keep the ratio (i.e., glutamate/aspartate-binding affinity) constant. Amplitudes are the optimized value plus 50 logarithmically distributed points between 1 and the ligand binding limit of 5 × 10^9^; binding rates are at -160 mV and normalized to 140 mM glutamate or aspartate. The same RSS was derived from a wide range of rate values, indicating that the KD determines the RSS and that individual binding/unbinding rate constants do not play a major role. (TIF) [file pcbi.1013214.s010.tif]

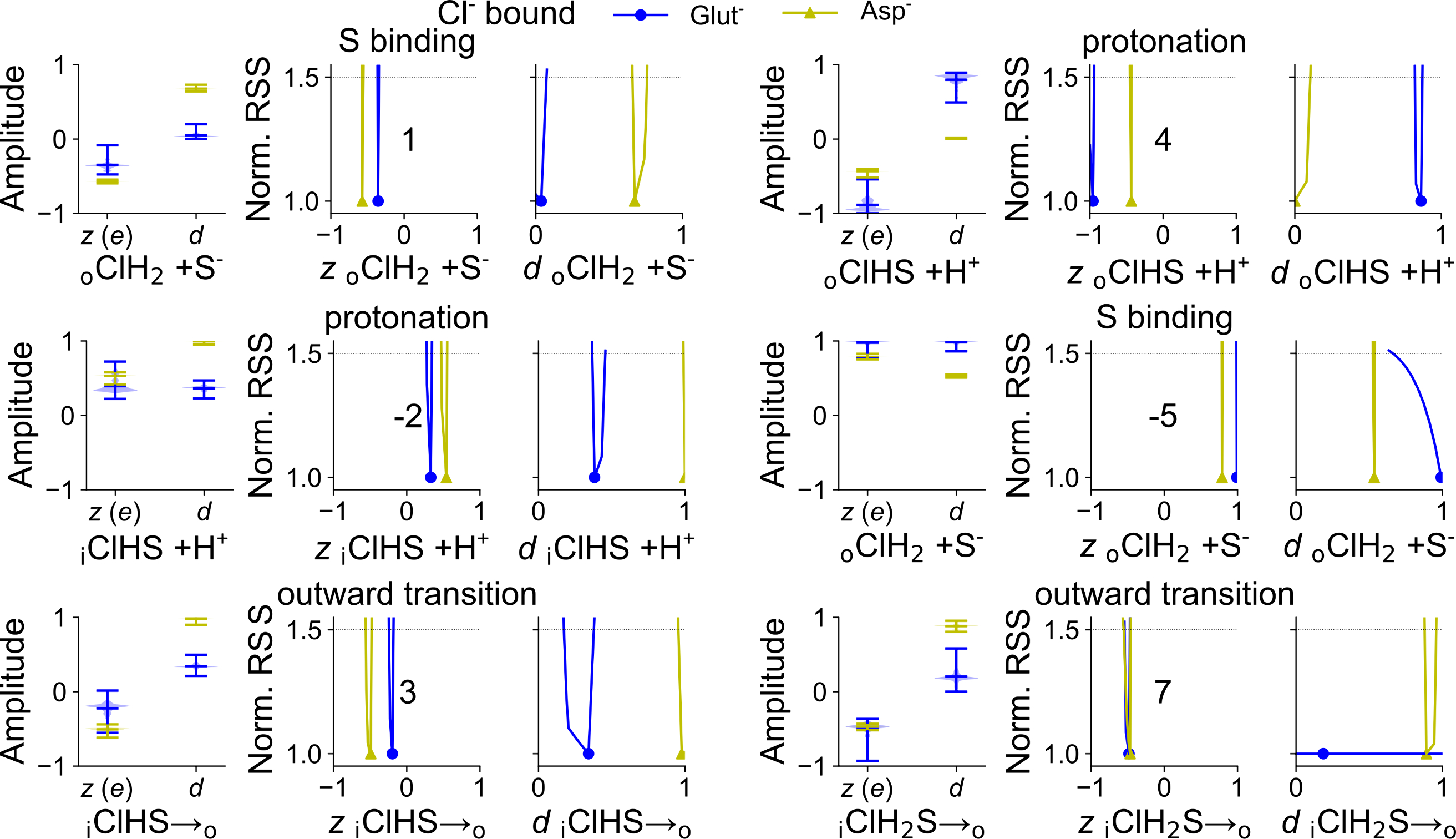

Supplement: S11 Fig — Distribution of z and d parameters for substrate binding (1 and 5), protonation steps (2 and 4), and transition between inward- and outward-facing conformations (3 and 7). Simulation results are given as violin plots depicting the amplitude range generated by exploratory mutation and normalized RSS representing goodness of fit for a range of amplitudes. Data for the outward transition of transport cycle step 6 (Fig 13) was omitted due to being substrate-independent. (TIF) [file pcbi.1013214.s011.tif]

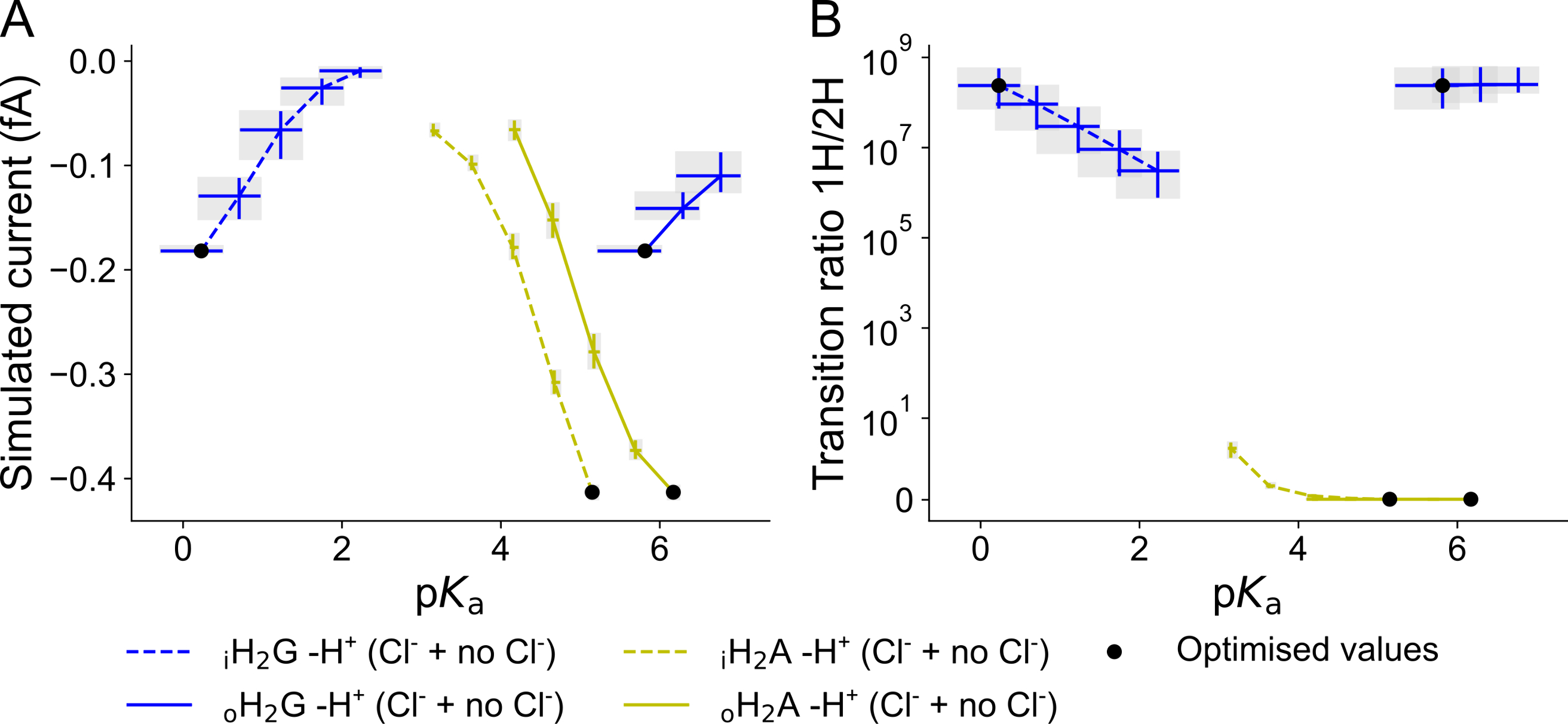

Supplement: S12 Fig — (A) predicted glutamate (blue) and aspartate (yellow) unitary currents (given as the number of charges per second in steady state × elementary charge) upon pKa modification. (B) relative number of H+-substrate exchange transport cycles upon pKa modification. The optimized pKa value for the second protonation in the inward- or outward-facing substrate-bound conformation is increased or decreased via the deprotonation rate constants by a factor of 3, 10, 33, or 100 while maintaining microscopic reversibility. Whereas the transport rates strongly depend on the pKa for both substrates, glutamate is transported in an exchange mode and aspartate in a uniport mode for all tested pKa values. (TIF) [file pcbi.1013214.s012.tif]

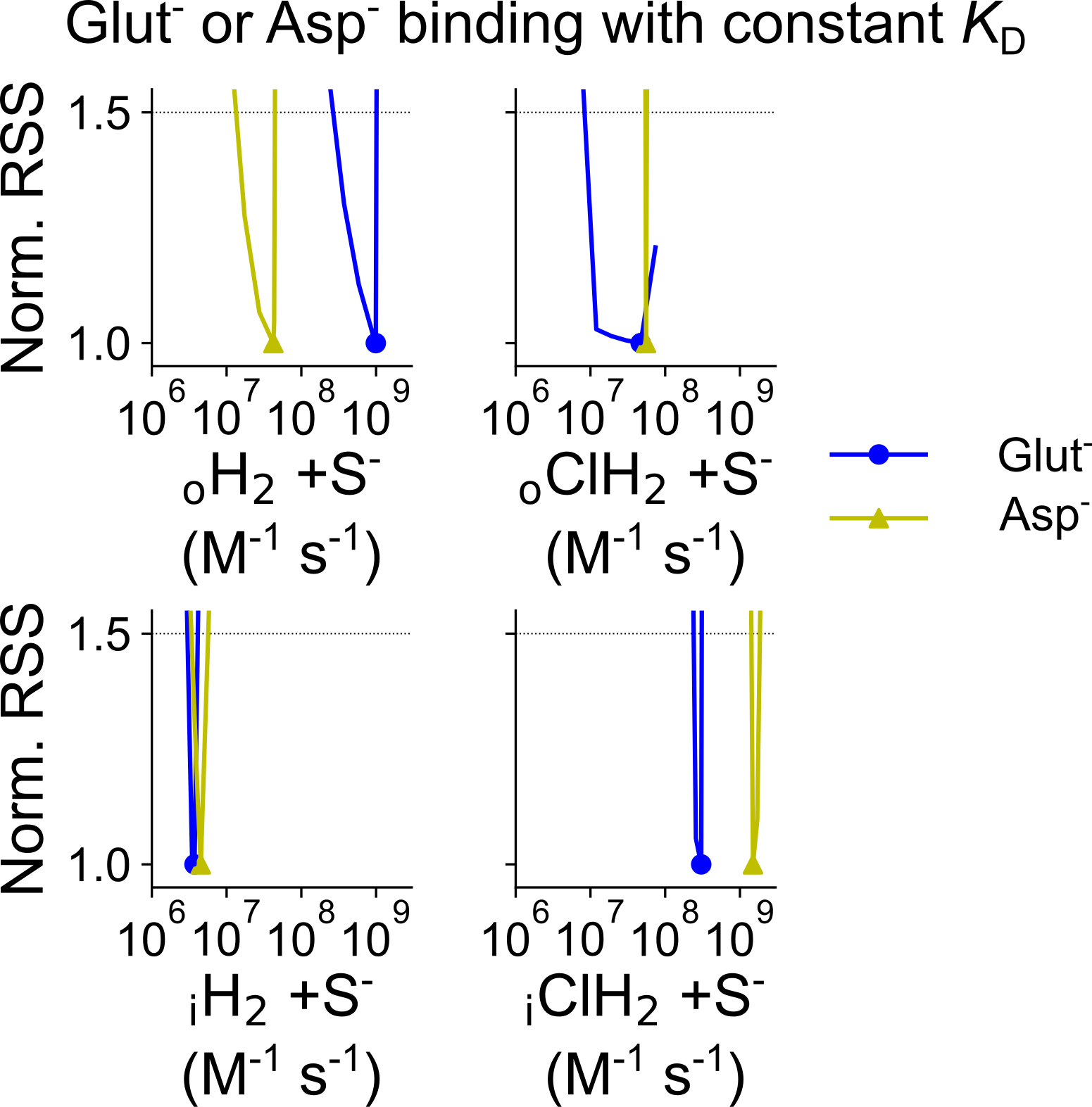

Supplement: S13 Fig — Statistical analysis for substrate binding rates, assuming constant KD. Changes in the goodness of fit upon modification of the binding rate for glutamate and aspartate, with and without Cl-, in both conformations. During modification unbinding constants were simultaneously altered to keep the ratio; i.e., the glutamate/aspartate binding affinity, unaltered. Amplitudes are the optimized value plus 50 logarithmically distributed points between 1 and the ligand binding limit of 5 × 10^9^, binding rates are at -160 mV and normalized to 140 mM glutamate or aspartate. The same RSS caused by a wide range of rate values indicates the KD determines the RSS, while individual binding/unbinding rate constants play no major role. (TIF) [file pcbi.1013214.s013.tif]

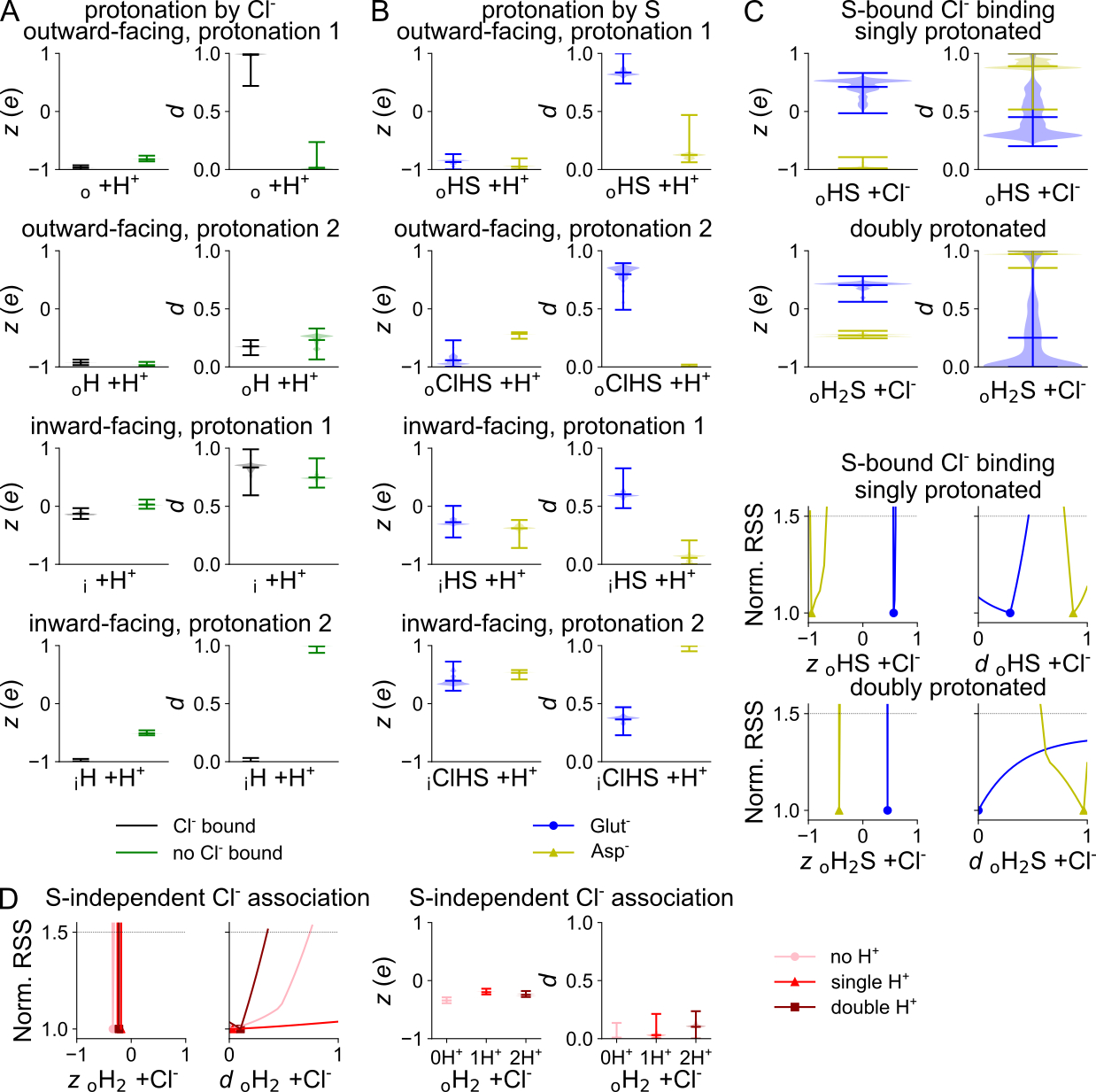

Supplement: S14 Fig — (A) parameters for the first and second protonation without bound glutamate or aspartate for inward- and outward-facing conformations, as modulated by external Cl-. (B) parameters for the first and second protonation with substrate bound for inward- and outward-facing conformations. (C) parameters for Cl- binding with substrate bound and with single or double protonation. (D) parameters for Cl- binding with no substrate bound and with no, single, or double protonation. Protonation parameters are represented by violin plots, other simulation results are given as normalized RSS representing goodness of fit for a range of amplitudes in addition to violin plots depicting the amplitude range generated by exploratory mutation. (TIF) [file pcbi.1013214.s014.tif]

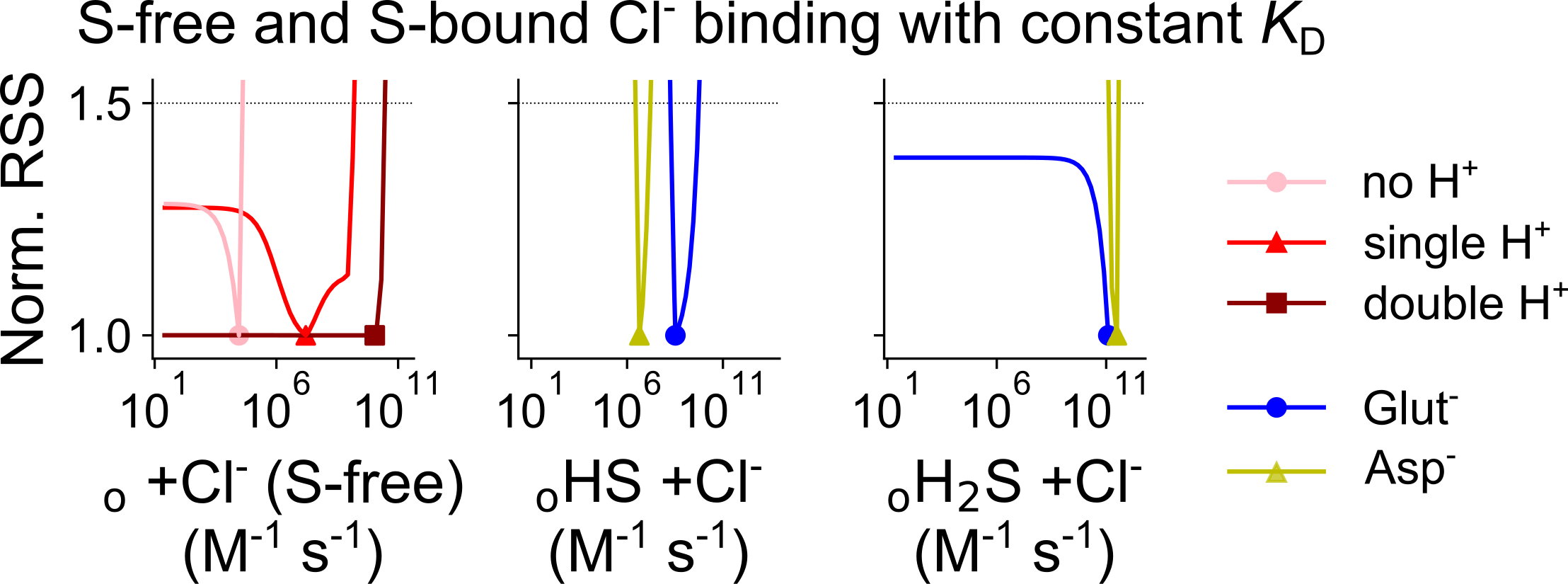

Supplement: S15 Fig — Changes in the goodness of fit upon modification of the Cl--binding rates with no substrate bound, by protonation state in shades of red (left), or with glutamate or aspartate (substrate) bound for the single and double protonation states (middle, right). During modification, unbinding constants were simultaneously altered to keep the ratio (i.e., the binding affinity) constant. Amplitudes are the optimized value plus 50 logarithmically distributed points between 1 and the ligand binding limit of 5 × 10^9^; binding rates are at -160 mV and normalized to a [Cl-] of 40 mM. The same RSS derived from a wide range of rate values indicates that the KD determines the RSS, with no major role for individual binding/unbinding rate constants. (TIF) [file pcbi.1013214.s015.tif]
